# Tribological Characterization of the Heat-Assisted Single Point Incremental Forming Process Applied to the Ti6Al4V Alloy with the Definition of an Adhesion Parameter for the Tool Surface

**DOI:** 10.3390/ma14247641

**Published:** 2021-12-11

**Authors:** Jesús Andrés Naranjo, Valentín Miguel, Juana Coello, María Carmen Manjabacas, Alberto Martínez-Martínez, Enrique García-Martínez

**Affiliations:** 1 High Technical School of Industrial Engineers of Albacete, University of Castilla-La Mancha, 02071 Albacete, Spain; jesus.naranjo@uclm.es (J.A.N.); juana.coello@uclm.es (J.C.); mcarmen.manjabacas@uclm.es (M.C.M.); Enrique.GMartinez@uclm.es (E.G.-M.); 2 Regional Development Institute, Science and Engineering of Materials, University of Castilla-La Mancha, 02071 Albacete, Spain; alberto.martinez@uclm.es

**Keywords:** HA-SPIF, Ti6Al4V, adhesion, surface finish

## Abstract

Heat-assisted single point incremental forming or HA-SPIF has a great potential for producing one-piece batches of hard-to-form materials such as Ti6Al4V alloy for medical and aeronautical applications. One of the limitations of the process is the difficulty in achieving a reasonable surface finish, which makes essential the characterization of the tribological process in the tool–sheet contact. In fact, not much work can be found at this point in literature. In this research, a novel procedure for evaluating the adhesion on the tool surface is proposed and the influence of the temperature is determined. The surface finish of parts is analyzed, and the changes promoted by HA-SPIF appearing in the morphology of the external surface layer are characterized by SEM.

## 1. Introduction

In spite of the potential applications of Ti6Al4V parts formed by SPIF, this alloy has limited formability at room temperature, and a heat-assisted technique must be applied in order to enhance this property [1,2,3,4,5,6,7,8,9,10,11,12,13,14].

Despite the improvement reached by heat-assisted SPIF (HA-SPIF) methods, the obtained surface finishes are of low quality. There is some research about the surface finish after SPIF process at room temperature, mainly not about titanium alloys. Thus, Lu et al. [15] proposed a variant in the SPIF tool for reducing the tool–aluminum sheet friction without lubricant to improve the surface finish of the part. Dakhli et al. [16] established that material sheet and lubricant are the most relevant factors in the optimization of surface finish by experimenting with AA1050 and Cu-Be2 alloys. Jawale et al. [17] researched SPIF of copper with lubrication and found that a tool of WC avoided adhesion during the forming. Nath et al. [18] experimented on AA7075 alloy with variable angle walls and established a worse surface finish for the greater forming; that is, the greater the angle, the higher the roughness parameters. Some researchers [19] focused their work on the evolution of tool wear; they considered a soft SPIF tool made of 189 HB steel for forming a galvanized steel.

Related to the influence of the SPIF process and its technological parameters applied to titanium alloys, Daleff et al. [20] experimented with SPIF on titanium F67 grade 2 at room temperature and obtained Ra values of 5.36 μm, on the face of the sheet in contact with the tool, with different SPIF parameter values. However, for biphasic α + β titanium alloys, for which the formability at room temperature is very poor, it would be relevant to study the surface finish after the HA-SPIF process, that is, try to analyze the influence of the process temperature on the surface finish of the produced parts. Recently, Vahdani et al. [21] carried out a study of the surface finish evolution with the use of Mo_2_S and graphite as lubricants. They applied a resistant heat system according to Joule’s law and obtained the best values of Ra, of about 1.50 μm, with graphite. Nevertheless, the temperature during the SPIF process was not controlled, but there were only two levels of electric current. In addition, the heating procedure is affected by the use of the lubricants appearing to sparkle. Ortiz et al. [22] planned the convenience of working at temperatures in the range of 650–700 °C to form the Ti6Al4V by SPIF. In their work, they reached a maximum temperature of 650 °C and obtained a bad finish surface basically due to the oxidative phenomena occurring during the process. Although these authors reinforce the viability of the HA-SPIF process applied to titanium alloys, they propose the need of applying post-SPIF surface treatments. Naranjo et al. [23] demonstrated the viability of using the HA-SPIF process with moderate temperatures from the viewpoint of the Ti6Al4V formability. These authors experimented with hardened steel and WC tools without lubrication and established that the friction process is in both cases predominantly adhesive, consisting of Ti6Al4V adhered on the tool surface [24]. In addition, the adhesion process of the Ti6Al4V alloy on the steel surface in friction processes has been confirmed in the literature [25,26]. Some authors even demonstrated the adhesive behavior on ceramic-type surfaces [27].

Taking into account what has been indicated above, the authors of the present work consider that there are limited reported data on the surface finish during the SPIF process. Particularly, studies on titanium alloys are few [21,24], and studies on the effect of the temperature on the surface finish are almost nonexistent [22]. For those reasons the present work achieves the following:

- Once the adhesive character tool-alloy during friction has been stated, a new parameter based on the material adhered to the tool surface is defined. The parameter consists of an average film thickness of the adhered material observed on the tool surface once the HA-SPIF process has finished.
- The influence of the temperature on the surface finish of Ti6Al4V parts obtained by the HA-SPIF process is characterized. A moderate temperature range, from room temperature to 400 °C, is covered.
- The changes in the morphology of the external surface layer promoted by the HA-SPIF process on Ti6Al4V parts are analyzed.

## 2. Methodology and Experimental Details

The experimental tests were run in a Deckel Maho machining center, model DMC 835V, with two different semispherical tungsten carbide tools of 10- and 12-mm diameter, respectively. SPIF was carried out by heating 0.8 mm thick Ti6Al4V alloy by a furnace. Assays were conducted at room temperature, 200, 300, and 400 °C without lubrication and taking care to fasten the sheet metal to a heated SPIF-fixture device just after the sheet had reached a uniform temperature. Thus, thermal stresses and/or deformations of the material during the heating process were avoided. To control the temperature during the SPIF process, a thermocouple located in the working area was connected to a controller and the temperature always remained at the setpoint +/−10 °C. More details about the SPIF system can be found in [23].

The forms executed were truncated cones with an outer diameter of 80 mm and either a constant or a variable wall angle. As in previous work [23], the parts with a variable wall angle are named funnel parts. The forming strategy adopted was a helical path with a pitch of 0.5 mm per revolution and a feed rate of 600 mm per minute. According to these conditions, the duration of the SPIF tests was within the range of 6–10 min depending on the total travel length of the tool. To minimize any friction between tool and sheet, a 30 rpm counter-rotation of the tool with respect to its displacement was set up. Each test was repeated twice.

**Figure 1:**
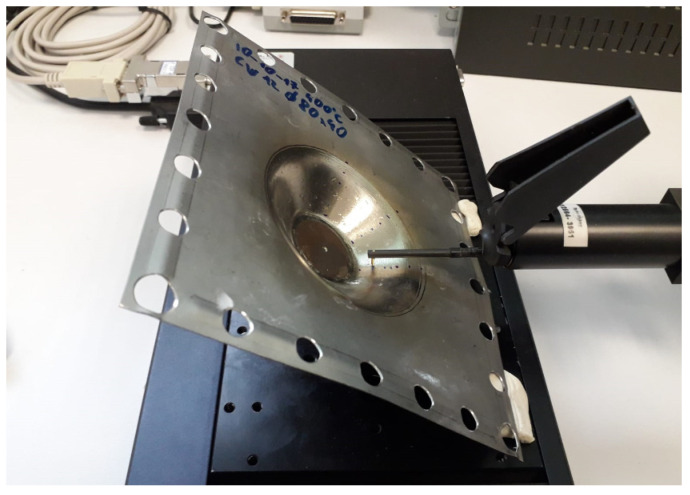
Profilometry experimental measuring of a part obtained by HA-SPIF.

After the SPIF tests, the roughness of the tool and the formed parts was measured with a Taylor Hobson Form Talysurf 50 roughness meter and treated with TalyMap Gold software (Taylor Hobson Ltd., LE4 9JQ, Leicester, UK), Figure 1.

**Figure 2:**
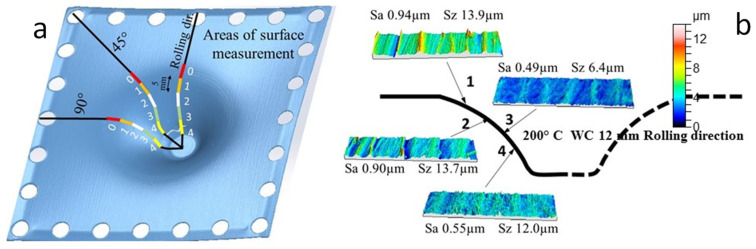
(**a**) Mapping design for measuring the roughness of SPIF parts; (**b**) results corresponding to a test with a 12 mm diameter WC tool at 200 °C.

The parts were measured in the directions of 0, 45, and 90° related to the rolling one. Three to five sections, depending on the depth, i.e., the formability of the cup made, were measured. As detailed in Figure 2, each measured area was 5 mm long by 2 mm wide. For removing the shape component in the measurements (variable wall cones), a grade 7 polynomial function was considered, since it allows the adjustment of different shapes, as established by some authors [28]. Finally, a 0.8 mm Gaussian filter was applied in order to obtain waviness and roughness profiles. For every two SPIF tests under the same conditions, the tool surface was ground with a 1000 grit SiC abrasive paper to remove the adhesive layers of titanium alloy on it.

## 3. Results and Discussion

### 3.1. Characterization of the Tool Surface Evolution

**Figure 3:**
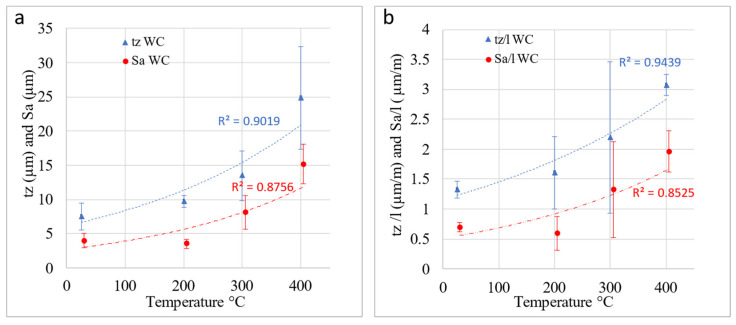
(**a**) The average arithmetic roughness Sa and material average thickness adhered on the tool surface, tz, after HA-SPIF tests; (**b**) Sa/l and tz/l. The values are obtained as a mean of the operations carried out with 10 and 12 mm WC tools. Error bars correspond to the maximum and minimum values obtained. The initial Sa value for WC tools was 0.15 μm.

For the selected sliding conditions, a severe titanium adhesive process on the surface of the tool was observed. Observation in setup tests revealed that the tool–sheet contact was modified right at the beginning of the process, i.e., adhesions of the Ti6Al4V appeared at the early stage of the process. The average arithmetic roughness, Sa, of the tool surface after the SPIF process at different temperatures is represented in Figure 3a. The results are expressed as the average of the values corresponding to the different tools experimented with. As can be seen, there is an acceptable correlation between Sa and the temperature of the SPIF process; Sa presents an increasing trend with the temperature. Sz values offered a great dispersion and have not been represented as they did not give any correlation and are, in this sense, a meaningless parameter.

The incremental formed sheet length was variable with the temperature because the greater the temperature, the higher the formability. Thus, all the SPIF tests carried out did not have the same tool–sheet sliding length, which varied from 1.5–5 m. Consequently, the parameter Sa has been normalized by dividing it by the total length, l, corresponding to each test. Figure 3b shows the values of Sa/l, although the correlations with the temperature are not better than those obtained for Sa.

To obtain a more representative parameter of the effect of the process on the tool surface, taking into account the evidence of the observed adhesion of sheet material to the tool surface, the equivalent thickness of the adhered layer was defined and evaluated. The adhesion phenomenon of the Ti6Al4V alloy on the steel surface in friction processes has been confirmed in literature [25,26,27]. There are also authors who demonstrated the adhesive behavior on ceramic-type surfaces [27], which reinforces the evidence obtained with the tools of WC tested in this study.

**Figure 4:**
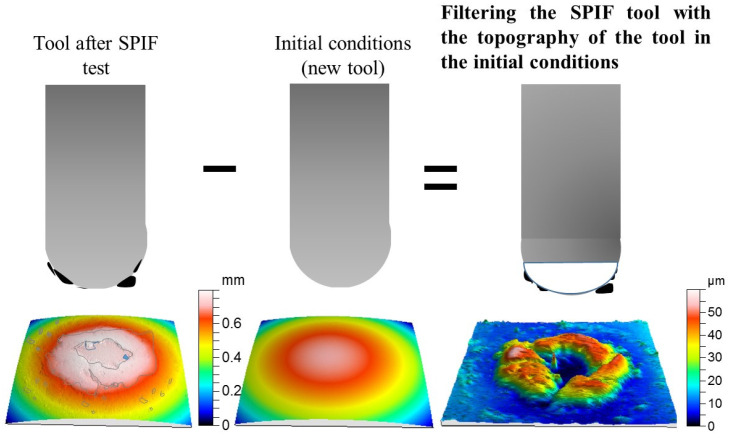
Sketch of the methodology for identifying the adhesion layer of Ti6Al4V adhered on the tool surface by unused tool filtering.

For that purpose, a new methodology was established. It consisted of subtracting the starting surface of the tool from the tool surface existing after the SPIF process; that is, the initial tool surface is removed from the tool surface with the Ti6Al4V adhesions as shown in Figure 4. The resulting surface is a direct consequence of the adhesion wear process on the tool surface, as well as the breakage of the film adhered to the tool in its contact with the sheet during the SPIF process. The passive oxide layer formed on the titanium adhered layer is only several nanometers thick and does not have a significant influence on this method. In addition, under SPIF conditions tested in this study, no relevant oxidation process takes place. The evidence observed allows us to state that there is hardly any wear on the tool, despite it having adhesive wear on the part with tribo-layer removal. In fact, this could be observed once the adhered layer was removed to restart the tool surface for a new test. The filtered surface represents the final state of the layer adhered to the tool, which will also be a function of its stability and the peel-out phenomenon taking place during the forming process. It is not necessary to use more filters (Gaussian, FFF, etc.) since the waviness has also been eliminated from the final surface by subtracting the initial one, whose forms are similar.

The subtraction of surfaces was carried out by the Talymap Gold software (Taylor Hobson Ltd., LE4 9JQ, UK), introducing both surfaces, the initial surface measured for the tool and the one corresponding to the state to be evaluated. The measurement of both surfaces was carried out while limiting the measurement area so that the overlap was maximum during the filtering or subtraction process. Moreover, the software allows the relative movement of one surface over the other to be done and shows the mean square error as an indicator of this overlap, which was minimized for each operation carried out.

**Figure 5:**
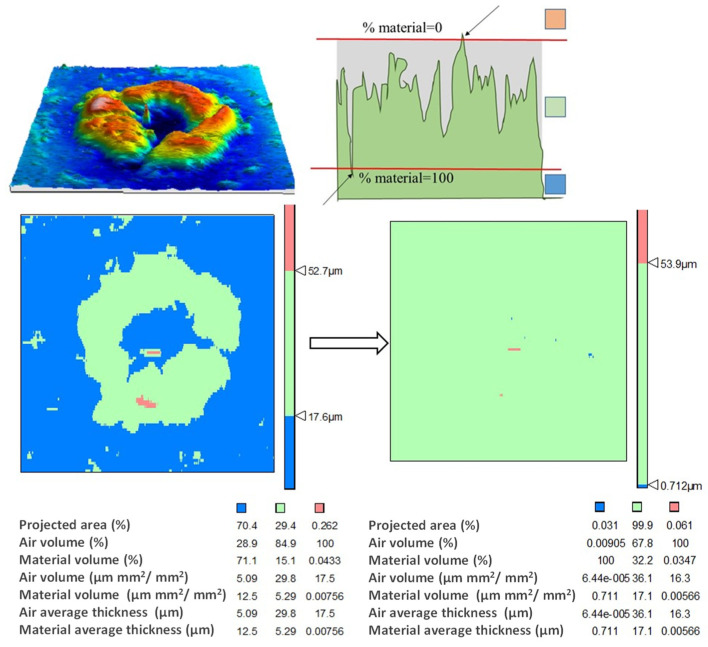
Methodology to evaluate the volume of adhered material on the tool surface.

After overlapping the surfaces and the subtraction operation, the software gave the results presented in Figure 5, which includes a 3D view of the surface and an adjustable diagram of the material surface. This diagram of the material surface allows two levels to be set up. The upper level corresponds to no material above it, 0%, and the bottom one indicates that only material exists below it, 100%. Finally, for both fixed levels, the software gave the values of the volume of existing material per unit of scanned area. That is equivalent to the average film thickness of adhered material and what constitutes the parameter known as tz shown in Figure 3. Logically, great care must be taken with the selection of the levels of material, particularly when there is some irregularity or adherence on the surface of very small volume, but which has a height far greater than the generality of the adhered surface. In these cases, locating the two levels of material distorts the estimation of the equivalent thickness of the adhered layer by disproportionately raising the maximum height of the surface. This circumstance is shown in Figure 5. Analogous to the treatment carried out with Sa, tz was divided by the length of displacement of the tool in each of the SPIF tests considered to obtain tz/l.

**Figure 6:**
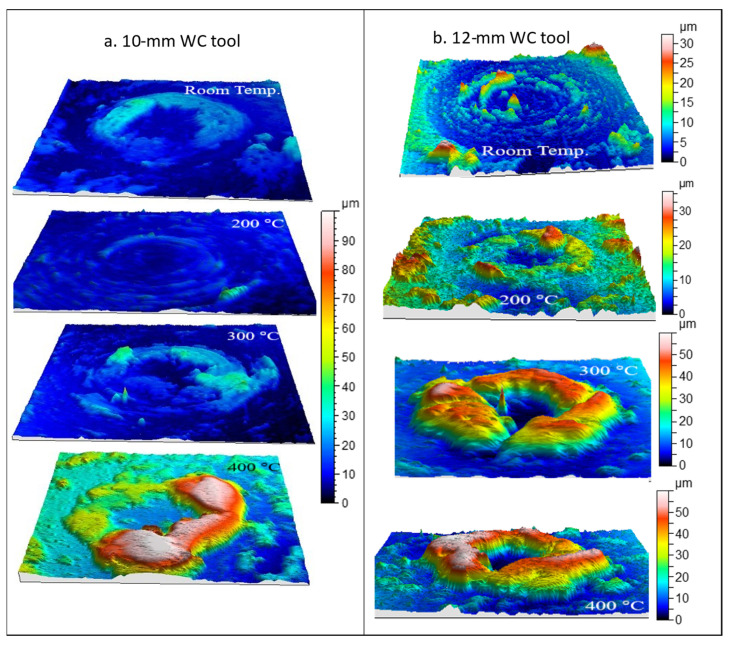
Adhered material on tool surfaces after SPIF at different temperatures with (**a**) 10 and (**b**) 12 mm WC tools. The results are similar in both cases, but the scale is uniform for 10 mm WC tool and optimized for every sample in (**b**).

Figure 6 shows the surfaces obtained by profilometry as indicated above. Qualitatively, strong material adhesion is observed on the surface of the tool, particularly in an area surrounding the tool tip. This area corresponds to that with the greatest pressure and contact with the sheet metal. It can be seen that there is a central zone, corresponding to the tip of the tool, where there is no contact with the sheet metal or the contact is made at very low pressure due to the rigidity of the material during the deformation process. This type of phenomenon has already been observed and quantified in sheet metal bending processes under tension [29].

The volume of adhered material generally increases with the forming temperature. At a quantitative level, the tz parameter allows the observed influence to be evaluated as depicted in Figure 3. A better correlation of the tz parameter with the temperature is observed when compared to that corresponding to Sa. Although a high correlation is also obtained for tz/l, it can be stated that the adhered layer thickness does not depend on the length friction significantly, because the correlations for tz and tz/l with the temperature are similar. The tz values are higher for temperatures above room temperature and are within the range of 7–25 µm. Therefore, the formation of an adhered layer does not evolve above this value (25 µm). All in all, some parallelism seems to exist between Sa and tz (also between Sa/l and Sz/l), which permits the authors to state that there is a quasidirect correlation between Sa and tz.

### 3.2. Variation of the Surface Finish of HA-SPIF Parts

**Figure 7:**
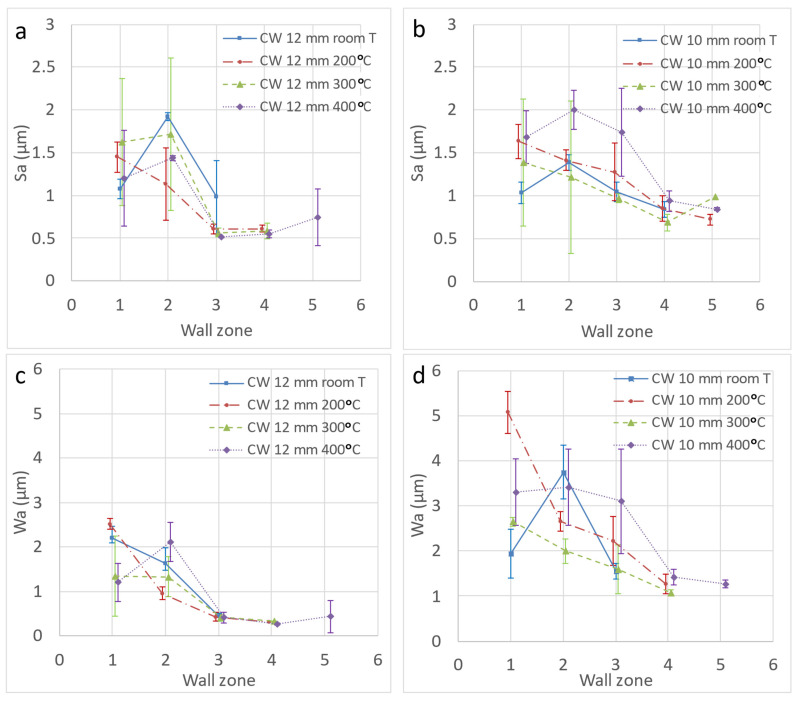
Sa and Wa surface parameters corresponding to the funnel parts obtained by HA-SPIF as a function of the wall zone (wall angle) and the temperature with a 12 mm WC tool (**a**,**c**) and with a 10 mm WC tool (**b**,**d**). Error bars correspond to the standard deviation of the measured values. The Sa value for the virgin sheet is 0.66 μm.

The variation of Sa in the different areas of the wall of the formed parts is not significant. The Sa values are higher in zones 1–2 in almost all cases, which can be attributed to the sheet bending that takes place just at the start of the forming process, where the friction is more irregular. From this point onwards, all tests lead to values of 0.5–1 µm. Again, the results for Sz were very dispersed and the only highlighted feature is that the maximum value measured was lower than 20 µm. In Figure 7a,b, Sa is represented for the different temperatures as a function of the zone on the wall of the funnel parts obtained by HA-SPIF. The results of Sa are worked out as the average value of the three measurements taken for each wall zone in the different areas of the part (0, 45, and 90° with respect to the rolling direction).

The values obtained for the waviness parameters, Wa and Wz, Figure 7c,d, are very similar to those for roughness. This means that the function used to eliminate the definitive shape of the wall, in the case of funnel parts, is adequate, confirming the choice of a grade 7 polynomial function as it is well suited to imperfect shapes. The low values of waviness obtained also establish that the forming process generates the part shapes in an adequate manner, with the elastic recovery of the material or springback being uniform throughout the formed product. That is, no peculiarities are established in terms of the wall section (wall angle) or the forming temperature. In short, it can be established that no local expansion or contraction occurs as a result of the plastic deformation carried out at a certain temperature. Again, the results correspond to the average value of the measurements indicated before.

Definitely, based on the roughness parameters analyzed, the temperature does not appear to have a negative influence on the surface finish of the processed sheet metal, despite what was observed in relation to the adherence of the alloy to the tool, being greater at higher temperatures.

**Figure 8:**
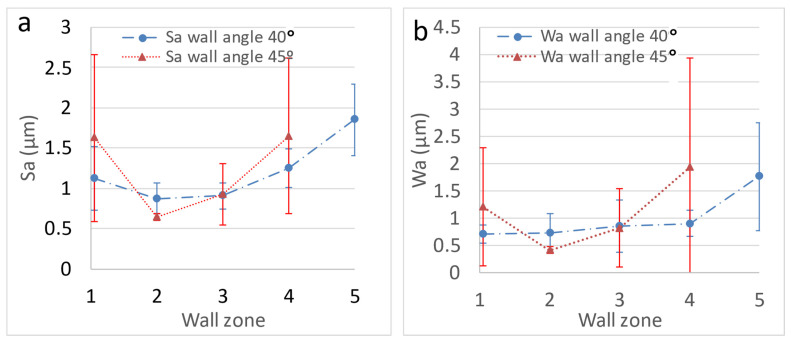
Sa (**a**) and Wa (**b**) surface parameters corresponding to conical parts (constant wall angle of 40 and 45°) obtained by HA-SPIF; tool: 12 mm WC. Error bars correspond to the standard deviation of the measured values.

On the other hand, the wall angle modifies the effective pitch of the horizontal movement of the sheet, given that the vertical one remains constant in the SPIF path of the tool; that is, the wall angle modifies the contact conditions. In this sense, it seems not to be of any influence on this circumstance, which can be confirmed if we compare the results obtained in constant wall cones with different wall angles. Thus, in Figure 8 the results corresponding to cones with 40 and 45° wall angles follow the same pattern.

### 3.3. Characterization of the Part Surfaces

**Figure 9:**
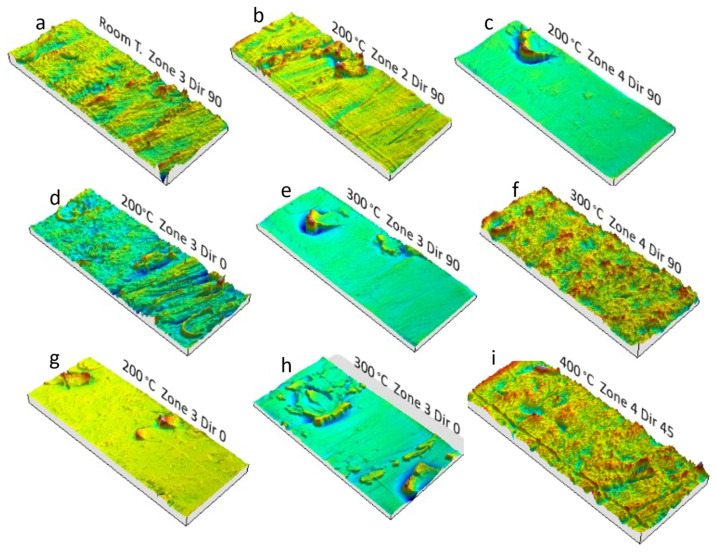
Surfaces of HA-SPIF funnel parts obtained from 3D profilometry analysis; 12 mm hardened steel. (**a**,**f**,**i**) Typical plowing phenomenon plus pitch effect and severe surface plastic deformation. (**b**–**e**,**g**,**h**) Local material accumulation by tool grooving.

**Figure 10:**
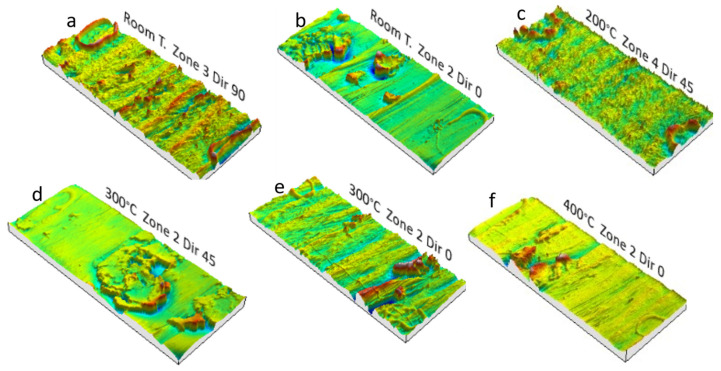
Surfaces of HA-SPIF funnel parts obtained from 3D profilometry analysis; 12 mm WC steel. (**a**,**c**) Plowing phenomenon and severe surface plastic deformation; some evidence of tool grooving. (**b**,**d**–**f**) Local material accumulation by tool grooving.

The 3D profiles obtained for several areas or wall angles and temperatures can also be analyzed. The generic pattern to which the different zones respond consists of material displacement and plowing with accumulations at certain points. Sometimes those phenomena are uniform and characteristic of the tool pitch during forming. In other tests, plowing is located around the tool, establishing promontories that remind us about the shape and diameter of the spherical tip of the tool, as can be seen in Figure 9 and Figure 10. Both situations are independent of the temperature and the wall angle, which justifies the high degree of dispersion of the values obtained for the Sz parameter.

**Figure 11:**
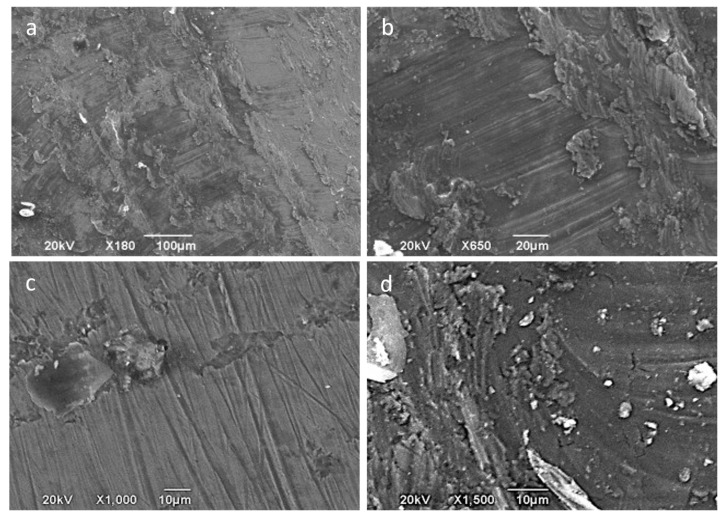
SEM macro- and micrographs (SE observation mode) corresponding to a funnel part processed at room temperature, central area of forming (2–3), tool of 12 mm hardened steel—φ 12 mm. (**a**) General view of the surface with the tribo-layer formed; (**b**,**a**) detail; (**c**,**a**) detail of peeling phenomenon; (**d**) material removal and adhesion of debris.

In spite of the high degree of adhesion existing between the friction surfaces, which leads to the migration of a significant layer of material on the tool surface, there are no large local pull-outs of material, which justifies the low values obtained for the average arithmetic roughness, Sa. Nevertheless, the analysis of the part surfaces by SEM reveals some uniform material removal produced on the surface of the sheet, as can be appreciated in Figure 11. This is a result produced by the wear and adhesive phenomena involved.

**Figure 12:**
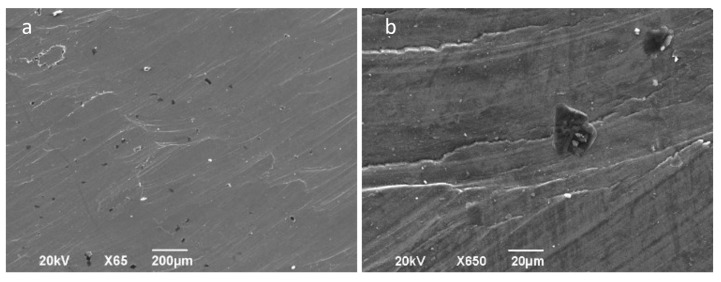
SEM macro- and micrographs (SE observation mode) of a part formed at 300 °C; central area of forming (2–3); tool of 12 mm hardened steel. (**a**) General view; (**b**) detail showing plastic deformation of the surface.

There is evidence of galling as shown in Figure 12a, characterized by the surface deformation lines that stand out. In Figure 12b an overlapping plastification of material can be observed, which reflects severe plastic deformation due to the high pressures in the sliding tool–sheet interface.

**Figure 13:**
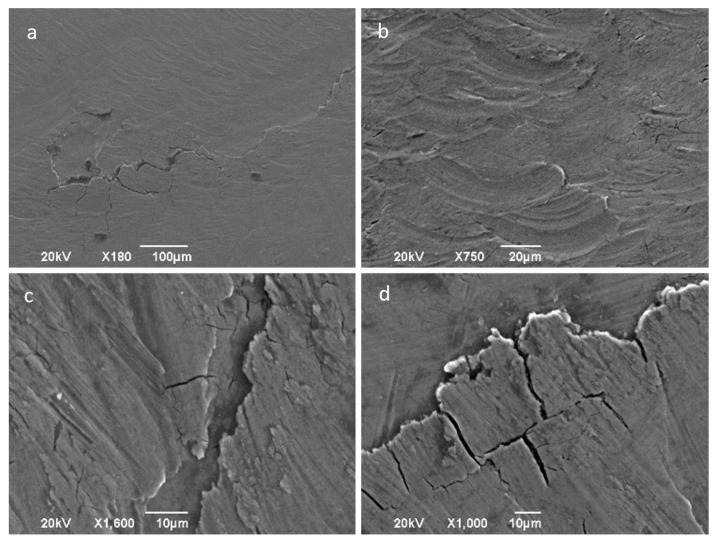
SEM macro- and micrographs (SE observation mode) corresponding to a part formed at 400 °C; central area (2–3); a and b with WC tool—φ 12 mm; c and d with hardened steel tool—φ 12 mm. (**a**) Cracks and delamination of surface area; (**b**) severe surface plastic deformation with material displacement; (**c**) detail of cracks and a narrow-delaminated area; (**d**) detail of cracks with incipient material being delaminated.

For the greatest deformations, reached with the highest temperature, 400 °C, the surface is characterized by a high state of cracking and material peeling as depicted in Figure 13a. A severe plowing or local deformation phenomenon is also observed in Figure 13b.

If roughness allows the surface finish of the material to be characterized, it is of great interest to analyze the evolution of the external oxidation layer of the Ti6Al4V sheet to provide information about the affection of the material surface during the SPIF process. It is known that titanium alloys have an oxidation layer that provides them with resistance to corrosion since it avoids the contact of the material with the medium. Several authors have established a low tribological quality of this layer [30].

**Figure 14:**
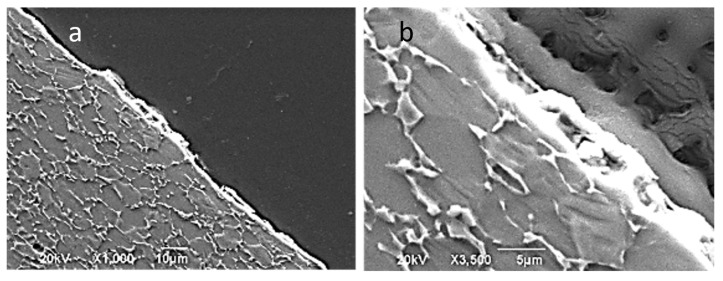
Tribo-layer of a cone of Ti6Al4V obtained at room temperature. (**a**) General view; (**b**) detail showing some debris particles embedded into the tribo-layer.

The evolution of the oxide layer in friction processes is also of great interest because it allows us to know the surface alteration that the material undergoes and even to make predictions of its tribological behavior in future friction processes. In titanium alloys subjected to friction, the surface oxide layer is, in general, partially eliminated by wear, although it regenerates very quickly. No layer delamination takes place as this usually occurs at very high friction speeds. At typical SPIF velocities, one of the wear mechanisms is the oxidative one [31]. In the face of the sheet in contact with the tool during the SPIF process, the formation of a tribological film or tribo-layer is also possible because of the generation of wear particles. These particles can be rejected from the contact zone of the friction surfaces, which affects the oxidation layer in the direction indicated above, i.e., layer wear and quasi-instantaneous regeneration. If particles remain in the contact zone or are not expelled, they can roll or slide between the contact surfaces, generating surface grooves and increasing the speed of wear. However, if particles remain fixed, they agglomerate in such a way that they create a tribological layer of particles or tribo-layer like the one observed in Figure 14 for a SPIF cone obtained at room temperature. Even at very high friction speeds, greater than 2 m/s, oxidation of the particles can occur, generating what some authors define as an oxidized tribological layer or “oxide tribo-layer” [32,33].

**Figure 15:**
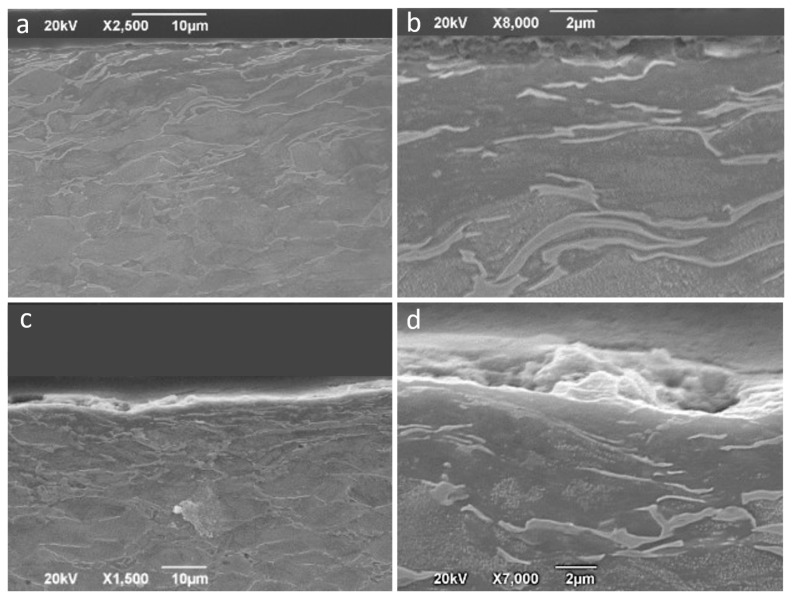
Tribo-layer of funnels of Ti6Al4V obtained at 200 °C (**a**,**b**) and at 400 °C (**c**,**d**). (**d**) Detail of the tribo-layer in which some existence of debris embedded can be appreciated.

Forming at 200 and 400 °C leads to a somewhat more uniform, but also irregular, tribological layer formation with r thicknesses of 1–2 µm at 200 °C as can be proven in Figure 15a,b. At 400 °C, layers with thicknesses of more than 3 µm are formed, as can be observed in Figure 15c,d. Figure 15 also allows us to observe the subsurface deformation of the sheet due to the mechanical action during the friction process. In the case of forming at 400 °C, the selected sample corresponds to the zone of maximum deformation or forming angle, and the thickness of the base material affected by friction is 5–10 µm.

## 4. Conclusions

The predominant wear mechanism in the HA-SPIF process of the Ti6Al4V alloy is of the adhesive type. The material is peeled out from the sheet metal and adhered on the tool surface. A direct correlation between the thickness of the material adhered to the tool and the duration of the test, i.e., the distance traveled by the tool in the process, has not been obtained. To explain that behavior, it is supposed a progressive growth of the adhered layer up to a maximum average thickness, for which it turns unstable and breaks. To characterize the adhesive phenomenon, a new parameter consisting of the average layer thickness on the tool surface has been proposed through a methodology based on the profilometry analysis. It has been found that the thickness of the material adhered to the tool increases with the forming temperature.

In relation to the formed parts, the roughness parameters Sa and Sz show high variability. This is explained by the material pull-out due to the strong adhesion phenomena indicated above. A plowing phenomenon is also noticeable, but no relevant abrasive wearing is observed as a quasi-single pass in the tool–sheet contact could be considered. Some of the particles worn or peeled out are trapped on the oxidized outer layer. Thus, tribo-layers that can change the tribology behavior of the workpiece in future applications are formed. The forming temperatures test do not appear to have an influence on the tribo-layers created.

## Data Availability

No new data were created or analyzed in this study. Data sharing is not applicable to this article.

## References

[1] Badr O.M., Rolfe B., Hodgson P., Weiss M. (2015). Forming of high strength titanium sheet at room temperature. Mater. Des..

[2] Göttmann A., Diettrich J., Bergweiler G., Bambach M., Hirt G., Loosen P., Poprawe R. (2011). Laser-assisted asymmetric incremental sheet forming of titanium sheet metal parts. Prod. Eng..

[3] Duflou J., Callebaut B., Verbert J., de Baerdemaeker H. (2007). Laser Assisted Incremental Forming: Formability and Accuracy Improvement. CIRP Ann..

[4] Fan G., Gao L., Hussain G., Wu Z. (2008). Electric hot incremental forming: A novel technique. Int. J. Mach. Tools Manuf..

[5] Fan G., Sun F., Meng X., Gao L., Tong G. (2009). Electric hot incremental forming of Ti-6Al-4V titanium sheet. Int. J. Adv. Manuf. Technol..

[6] Fan G., Gao L. (2014). Mechanical property of Ti-6Al-4V sheet in one-sided electric hot incremental forming. Int. J. Adv. Manuf. Technol..

[7] Magnus C.S. (2017). Joule heating of the forming zone in incremental sheet metal forming: Part 1. Int. J. Adv. Manuf. Technol..

[8] Magnus C.S. (2017). Joule heating of the forming zone in incremental sheet metal forming: Part 2. Int. J. Adv. Manuf. Technol..

[9] Honarpisheh M., Abdolhoseini M.J., Amini S. (2015). Experimental and numerical investigation of the hot incremental forming of Ti-6Al-4V sheet using electrical current. Int. J. Adv. Manuf. Technol..

[10] Ambrogio G., Filice L., Gagliardi F. (2012). Formability of lightweight alloys by hot incremental sheet forming. Mater. Des..

[11] Francesco G., Giuseppina A., Luigino F. (2017). Incremental Forming with Local Induction Heating on Materials with Magnetic and Non-magnetic Properties. Procedia Eng..

[12] Durante M., Formisano A., Langella A., Minutolo F.M.C. (2009). The influence of tool rotation on an incremental forming process. J. Mater. Process. Technol..

[13] Palumbo G., Brandizzi M. (2012). Experimental investigations on the single point incremental forming of a titanium alloy component combining static heating with high tool rotation speed. Mater. Des..

[14] Göttmann A., Bailly D., Bergweiler G., Bambach M., Stollenwerk J., Hirt G., Loosen P. (2013). A novel approach for temperature control in ISF supported by laser and resistance heating. Int. J. Adv. Manuf. Technol..

[15] Lu B., Fang Y., Xu D., Chen J., Ou H., Moser N., Cao J. (2014). Mechanism investigation of friction-related effects in single point incremental forming using a developed oblique roller-ball tool. Int. J. Mach. Tools Manuf..

[16] Dakhli M., Boulila A., Manach P.-Y., Tourki Z. (2019). Optimization of processing parameters and surface roughness of metallic sheets plastically deformed by incremental forming process. Int. J. Adv. Manuf. Technol..

[17] Jawale K., Duarte J., Reis A., Silva M.B. (2018). Microstructural investigation and lubrication study for single point incremental forming of copper. Int. J. Solids Struct..

[18] Nath M., Shin J., Bansal A., Banu M., Taub A. (2018). Comparison of Texture and Surface Finish Evolution During Single Point Incremental Forming and Formability Testing of AA 7075. TMS Annual Meeting & Exhibition.

[19] Da Silva P.J., Alvares A.J. (2019). Investigation of tool wear in single point incremental sheet forming. Proc. Inst. Mech. Eng. Part B J. Eng. Manuf..

[20] Daleffe A., Schaeffer L., Fritzen D., Castelan J. (2013). Analysis of the Incremental Forming of Titanium F67 Grade 2 Sheet. Key Eng. Mater..

[21] Vahdani M., Mirnia M.J., Gorji H., Jooybari M.B. (2019). Experimental Investigation of Formability and Surface Finish into Resistance Single-Point Incremental Forming of Ti–6Al–4V Titanium Alloy Using Taguchi Design. Trans. Indian Inst. Met..

[22] Ortiz M., Penalva M., Iriondo E., de Lacalle L.N.L. (2019). Accuracy and Surface Quality Improvements in the Manufacturing of Ti-6Al-4V Parts Using Hot Single Point Incremental Forming. Metals.

[23] Naranjo J.A., Miguel V., Martínez A., Coello J., Manjabacas M.C. (2019). Evaluation of the Formability and Dimensional Accuracy Improvement of Ti6Al4V in Warm SPIF Processes. Metals.

[24] Naranjo J., Miguel V., Martínez A., Coello J., Manjabacas M., Valera J. (2017). Influence of temperature on alloy Ti6Al4V formability during the warm SPIF process. Procedia Eng..

[25] Long M., Rack H. (2001). Friction and surface behavior of selected titanium alloys during reciprocating-sliding motion. Wear.

[26] Bansal D., Eryilmaz O., Blau P. (2011). Surface engineering to improve the durability and lubricity of Ti–6Al–4V alloy. Wear.

[27] Findik F. (2014). Latest progress on tribological properties of industrial materials. Mater. Des..

[28] Le Mercier K., Dubar M., Mocellin K., Dubois A., Dubar L. (2017). Quantitative analysis of galling in cold forging of a commercial Al-Mg-Si alloy. Procedia Eng..

[29] Martínez A., Miguel V., Coello J. (2018). A new approach to evaluate bending forces for deep-drawing operations of a TRIP700 + EBT steel sheet. Int. J. Mater. Form..

[30] Budinski K.G. (1991). Tribological properties of titanium alloys. Wear.

[31] Straffelini G., Molinari A. (1999). Dry sliding wear of Ti–6Al–4V alloy as influenced by the counterface and sliding conditions. Wear.

[32] Zhou Y., Wang S., Chen W., Jiang W., Wang L., Chen K., Cui X. (2018). Comparative research on the effect of an oxide coating and a tribo-oxide layer on dry sliding wear of Ti–6Al–4V alloy. Proc. Inst. Mech. Eng. Part J J. Eng. Tribol..

[33] Zhang Q., Ding H., Zhou G., Guo X., Zhang M., Li N., Wu H., Xia M. (2018). Dry Sliding Wear Behavior of a Selected Titanium Alloy Against Counterface Steel of Different Hardness Levels. Met. Mater. Trans. A.

